# Technology for deep brain stimulation at a gallop

**DOI:** 10.1002/mds.26253

**Published:** 2015-05-23

**Authors:** Alberto Priori

**Affiliations:** ^1^ Clinical Center for Neurostimulation, Neurotechnology and Movement Disorders, Fondazione IRCCS Ca' Granda Ospedale Maggiore Policlinico, and University of MilanItaly

Along with levodopa and botulinum toxin, deep brain stimulation (DBS) has been a milestone achievement in the treatment of movement disorders. Although the hardware for DBS has undergone technological improvement, and more than one manufacturer has appeared in the global market, in general the concept for DBS has remained almost unchanged over the past two decades. As the pathophysiology of movement disorders becomes clearer (thanks partly to DBS itself) and electronic know‐how and equipment advance, an even more fascinating future opens for treating patients. As happens in other therapeutic fields, not every innovation brings about substantial changes that will really influence patients' quality of life, but many can simply involve commercial restyling aimed to push the market forward. Technological advancements have an especially important role in DBS, now becoming an increasingly more appealing business given the growing Parkinson's disease (PD) prevalence—expected to reach 8.7 million in 2030^1^—and the evidence that early DBS implant induces benefits.^2^ Currently the estimated market for DBS devices for PD is approximately $200 million to $300 million per year worldwide, but the coming years promise a further powerful push.^3^

Because good clinical practice requires us to offer our patients the best possible and most suitable healthcare solutions, we should critically assess whether technical advances are really useful. To provide clinicians with the knowledge needed to orient themselves in the wide innovative DBS field, here I introduce and try to explain in simple terms understandable to the general reader DBS technological advances that could improve health care. In very broad terms, the most relevant recent technological progresses in the DBS field outlined later aim to increase the temporal and spatial resolution of this treatment by developing solutions to deliver stimulation only where and when it is needed, thus reducing energy wasting and limiting adverse effects.

## Technology Implanted Into the Patient

### A New Approach: Adaptive DBS (aDBS)

Movement disorders are typically fluctuating conditions. The best example is obviously PD, in which patients in the advanced stages undergo within minutes remarkable clinical changes ranging from complete motor blocks to severe dyskinesias. Conventional DBS (cDBS) delivered with constant parameters, regardless of the individual patient's clinical state, can lead to suboptimal symptomatic control. Because several DBS adverse effects, including dyskinesias, can be reversed by reprogramming DBS parameters,^4^, ^5^, ^6^ aiming to investigate ways to improve DBS outcome, researchers some 10 y ago began developing “intelligent” devices that could automatically adapt stimulation parameters moment‐by‐moment to the patient's clinical state. This novel approach, termed adaptive DBS (aDBS), is based on closed‐loop systems using a control signal captured through a sensor. The signal is then fed into a controller circuit, which in turn adapts DBS settings. Although all of the proposed adaptive systems have this general structure, several theoretical approaches exist according to the type of control signal used and parameters the system adjusts.^7^

**Figure 1:**
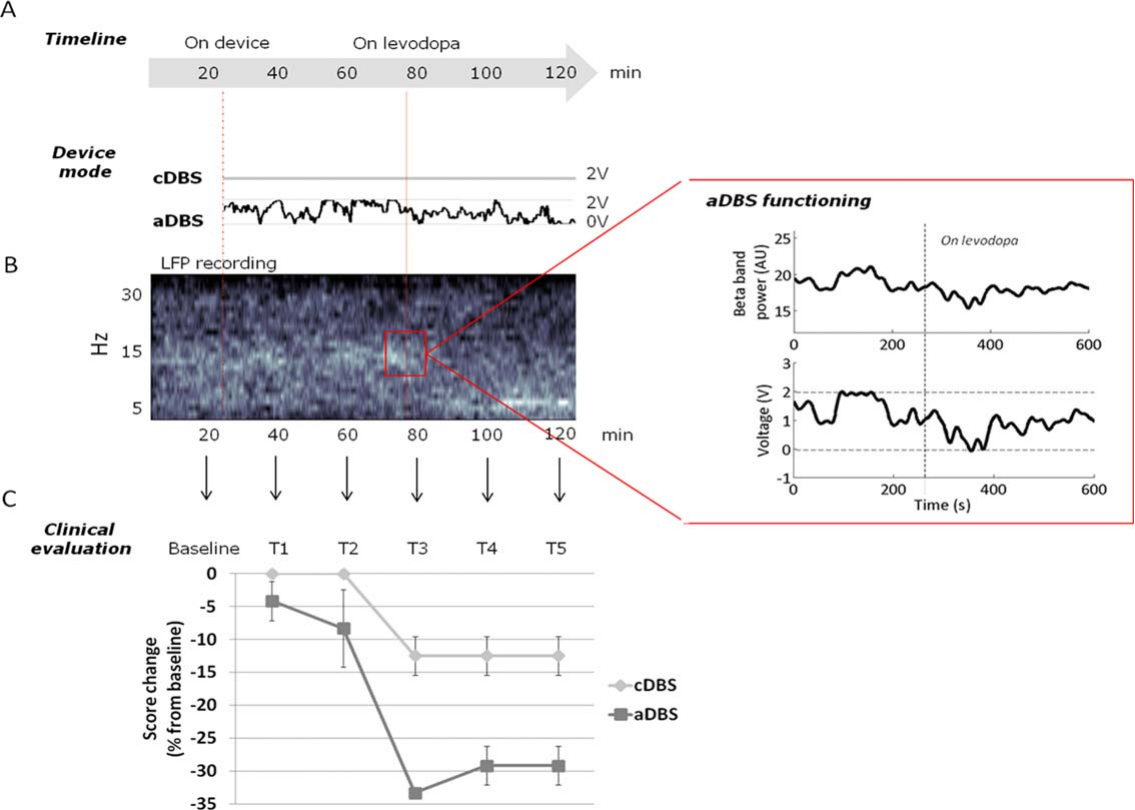
Adaptive STN deep brain stimulation in a freely moving patient with Parkinson's disease. (man, age:51 y,disease onset: 8 y) (**A**) The arrow at the top represents the timeline for the experimental sessions (120 min) taking place 5 and 6 d after electrode implantation to test the clinical effectiveness of conventional DBS (cDBS) and adaptive DBS (aDBS). The first dotted line represents the time when the device is turned on (after the baseline clinical assessment), and the second line (at about 80 minutes) the time levodopa took to achieve its clinical effect. Below the DBS voltages for the two device modes: the cDBS voltage is 2 V throughout the experimental session, whereas aDBS voltage changes according to the online analysis of local field potential (LFP) recordings as also shown in **B** on the left (time‐frequency plot for LFP power). (**B**) In the expansion on the right, the aDBS functioning sample lasting 600 sec: the voltage delivered by aDBS “followed” the beta‐band changes: when levodopa reduced beta‐band LFP activity, the voltage diminished. (**C**) Clinical result. The plot shows the percentage changes from baseline for bradykinesia items of Unified Parkinson's Disease Rating Scale motor part subsection (items 23, 24, 31) for aDBS and cDBS evaluated every 20 min by two blinded neurologists From Rosa et al. 56. (from T1 to T5). Note that aDBS clearly improves the motor score more than cDBS (Modified from 56).[Color figure can be viewed in the online issue, which is available at wileyonlinelibrary.com.]

The aDBS approach now in the most advanced study phase in humans uses as control signals local field potentials (LFPs) recorded through the same electrode used for DBS.^8^ Local feld potentials are biopotentials (closely resembling the electroencephalogram signal) recorded from the target brain structure. They represent summated local electrical current and are generally thought to arise from synaptic input activity. In PD they can reflect the patients' clinical state.^9^ Schematically, the increased power in the beta frequency band (8‐35 Hz) correlates with akinesia^10^ or freezing of gait,^11^ the low‐frequency oscillations correlate with dyskinesias and behavioral disturbances (i.e., impulsivity) related to dopaminergic stimulation,^12^, ^13^ and the gamma (30‐48 Hz) oscillation is thought to have a prokinetic role.^14^ An important technological advance that makes LFPs a good candidate as a robust control signal came from research developing a system able to record LFPs during ongoing DBS and deliver stimulation through the same electrode.^15^ Hence, according to the individual patient's LFP oscillation pattern, the low‐frequency oscillations, or the beta band, or the gamma band in subthalamic LFP activity can be used alone or together as signals to control DBS automatically. Although the inventory of LFP oscillations in patients with movement disorders has remarkably increased, we still know little about which among the various frequencies provides the pathophysiological key to PD or to the DBS‐induced therapeutic effects. No matter whether the discovered brain oscillatory patterns (including the increased beta power in LFPs) are merely an epiphenomenon, a biomarker pointing to a pathophysiological mechanism, or an ascertained mechanism, they can still be technically useful as control signals for aDBS. The absence of increased beta power in LFPs in approximately one third of the patients^16^, ^17^ proves that these oscillations are not invariably a pathophysiological biomarker. A recent report confirmed the idea that aDBS provides better symptomatic control than cDBS in a small group of patients with PD,^18^ and ongoing clinical trials are now comparing aDBS with cDBS in patients with PD (Fig. 1). The availability of portable external devices for aDBS that can be used to study freely moving patients for hours^19^ will help to establish whether and how adaptive strategies can really improve patients' day‐to‐day lives. Despite these encouraging preliminary data, no systematic studies investigating the underlying neuronal physiology yet support the notion the aDBS is more effective than cDBS. Although Little et al.^18^ reported that beta oscillation–controlled aDBS has a greater clinical effect than cDBS on patients' motor assessments, extrapolating this finding to the underlying neuronal mechanisms would be misleading. Equally important, beta‐controlled aDBS obviously cannot be used to treat patients without beta oscillations.

Another control signal proposed for use in DBS in humans is the tremor signal captured through external sensors measuring surface electromyography (sEMG) and accelerations.^20^ These signals, analyzed through spectral and nonlinear techniques, are used to trigger DBS onset for suppressing pathological tremor. This strategy is still in an experimental phase^20^ and is limited by the need for additional hardware (surface electromyography and accelerometers) that can restrict the patient's activities.

As a possible control variable for adapting DBS settings, others have proposed and tested in animals neurochemical signals.^21^ This idea grounds on the hypothesis that DBS evokes changes in neurotransmitter release. Thus, characterizing DBS‐induced neurochemical changes can help in defining optimal therapeutic stimulation settings, especially for an aDBS strategy in psychiatry. The system has already been tested in a rodent PD model^18^ designed to adjust stimulation settings according to changes in brain dopamine levels.

Whatever the control signal, once it is fed into the controller device, several different options exist for modulating DBS: in relation to the patient's clinical need, the system can automatically adapt the stimulation site (changing the contact on the same electrode or even using an electrode in a different target structure) or the stimulation settings (including voltage, intensity, frequency, waveform, and polarity), or changes in site and settings combined. Even though the effects induced by manipulating stimulation sites or settings for an aDBS device remain unexplored and should be systematically tested in future research with novel devices, data from cDBS offer hints for future research directions. For instance, besides the obvious effects induced by stimulation voltage or current, another variable suitable for automatic adaptation might be stimulation frequency: subthalamic DBS at 60 to 80 Hz can in some patients provide better control over axial symptoms, speech, and dyskinesias,^22^ and DBS delivered with personalized frequencies can effectively reduce Unified Parkinson's Disease Rating Scale motor scores.^23^ Also, different frequencies could be automatically set during wakefulness or during sleep. The rationale for changing stimulation frequency could be that because the human basal ganglia operate in the amplitude modulation and frequency modulation modes,^24^ the two modes for adapting stimulation (modulating its amplitude in the amplitude modulation mode, or modulating its frequency in the frequency modulation mode) can interact with basal ganglia processing in these two informational domains, thus theoretically doubling the DBS‐induced effects.

A final promising advance is that of equipping novel aDBS systems with algorithms that “learn” how to optimize the stimulation strategy in individual patients. Because clinicians know how each patient differs from others, another major aim is to design systems that maximize DBS benefits by auto‐setting best stimulation according to the individual patient's characteristics.

The next step in the aDBS field is to develop an implantable device, but before achieving this aim research has to overcome several hurdles. Among technological advancements are implantable systems that can record and process neurophysiological data to detect the individual patient's clinical state and automatically adjust stimulation accordingly. Whereas personal computer–based analysis in the laboratory^18^ can extract such information regardless of the computational effort, in implantable devices power consumption and device dimensions both strongly limit processing possibilities. To overcome these limitations, advances in microelectronics for implantable neurostimulators have produced miniaturized solutions for low‐power, low‐noise neural recording.^25^ In DBS applications, neural sensing is further complicated by the need for suppressing the stimulation artifact. Although neural signals can be concurrently recorded and stimulation delivered on an external device,^15^ available integrated circuits for implantable devices^26^, ^27^, ^28^ only partially address the problem. Another key technical issue in developing aDBS is the need for continuous LFP signal analysis. Because software programs for continuous spectral analysis in real‐time entail prohibitively high energy consumption, some have proposed dedicated analog and digital hardware architectures for extracting information from neural signal recordings.^29^, ^30^, ^31^, ^32^ Integrated circuit solutions, integrating low‐power recording and processing capabilities, novel battery technology, and the advent of rechargeable batteries will boost the development and availability of aDBS devices.

The economic burden of aDBS strategies is difficult to estimate. Nonetheless, among its expected advantages, this new technique promises to prolong battery life, thus reducing the frequency of surgical procedures required for its replacement (and the related risks). Because a German study^33^ showed that DBS costs arose mainly from battery exchange, prolonged battery life would presumably decrease treatment costs.

### Interleaving Stimulation, Current Steering, and Dual Stimulation

Rather than being a novel concept, current steering achieved by manually selecting a contact has been used since DBS was first introduced and has now been refined through specific technological solutions that should allow optimal shaping of the electrical fields in the brain, thus tailoring stimulation to the individual patient's needs. Two developments that have advanced the original concept of steering are interleaving and current steering in the plane orthogonal to the long axis of the DBS lead.

Interleaving stimulation entails delivering a varying charge amount through different contacts on the same DBS electrode, stimulating at varying stimulation strengths and pulse widths in a temporally alternating sequence but at the same frequency. Because this approach allows the system to optimize the electric field in time and in space (in some cases according to specific software simulations estimating the activated tissue volumes), it induces fewer adverse effects. Few reports describe effectiveness of interleaving protocols in selected patients with PD,^34^, ^35^ essential tremor,^36^ and dystonia^37^ with a nonoptimal response to conventional DBS protocols. Research now needs to make programming less expensive, decrease energy consumption so as to lengthen battery life, find ways to use independent frequencies, and seek systematic data on whether interleaving stimulation is effective. Little evidence yet shows that an interleaving program is useful for most patients.^38^

Current steering in general refers to concurrently delivering different current strength**s** through different contacts on the same DBS electrode, implying that the electric field can be steered either along the longitudinal axis, or directionally around the longitudinal axis, or together along and around at the same time. Simulation software can be used to model the shape of the induced electrical field. Current steering along the longitudinal axis using a system able to reshape the electrical field along the four electrode contacts and fractionalize the current has been reported to be effective in a PD patient responding poorly to a standard DBS protocol.^39^ Another technical approach allowing current steering around the longitudinal axis uses directional electrodes. Currently available electrodes for DBS have cylindrical contacts generating an electric field at 360 degrees and omnidirectional stimulation. Theoretically, the use of directional electrodes that can deliver a stimulation‐oriented and radial current steering toward a specific structure can increase the therapeutic window for DBS by reducing the adverse effects. Using specifically designed electrodes in 11 patients with PD and 2 with essential tremor, Pollo et al.^40^ intraoperatively tested three different stimulation directions and omnidirectional stimulation. They found that compared with omnidirectional stimulation directional stimulation significantly widened the therapeutic window and lowered the current strength needed for beneficial effects. Even though the study reports only intraoperative data and needs to be confirmed in long‐term follow‐up, the findings clearly suggest directional stimulation as another strategy to obtain better DBS responses. Obviously combining technical solutions allowing current steering along and around the electrode's longitudinal axis would theoretically bring about further improvements, shaping the electric field in a multidirectional way. Under this hypothesis, a 32‐contact electrode for DBS able to steer the electric field longitudinally and radially has been proposed and preliminarily tested in patients.^41^, ^42^ A possible drawback to this solution is probably the time‐consuming programming needed for a system with 32 contacts. These systems might benefit from automatic stimulation settings.

Finally, DBS could be simultaneously delivered in two different brain structures with independent stimulation settings from any of the 16 electrode contacts, ultimately leading to dual stimulation. For instance, stimulation delivered to the thalamus at 132 Hz and the periventricular gray area at 10 Hz has been used in a case of phantom limb.^43^

### Constant Current Stimulation

The nervous system can be electrically stimulated in two modes: keeping the voltage (Volt or millivolt) constant, or keeping the current intensity (Ampere or milliampere) constant. Whereas at a fixed voltage changes in the electrode–brain interface alter the stimulus current level and, in turn, the charge exchanged with the tissue, constant‐current stimulation produces an adjustable current through the electrode–brain interface independent from its impedance. The presence of capacitive components in the electrode–brain interface means that the current pulse induced in the brain by the two stimulation modes differs in shape and, possibly, also in effects. Like current‐steering technology, constant‐current stimulation has for decades been preferred to constant voltage stimulation in neurophysiology. The DBS era began with a constant voltage approach simply because the first device was developed from a cardiac pacemaker. Although the technology and the hardware differ between constant‐voltage and constant‐current stimulation, constant current provides a more accurate control over stimulation, and is especially useful in conditions in which the impedance can change, such as DBS.^44^ Most commercially available DBS devices deliver constant‐voltage stimulation. Seeking more information on constant‐current DBS, Okun and Foote^43^ studied a group of 136 patients with PD implanted with a device delivering constant‐current stimulation. Even though the study was unblinded and provided no direct comparison with constant‐voltage stimulation, after patients received constant‐current stimulation their symptoms improved, and adverse effects and complications overlapped those reported in the literature for constant‐voltage stimulation. Despite these promising findings, no proven physiological or clinical rationale exists for choosing constant‐current versus constant‐voltage or vice versa, and both approaches provide similar results.^45^ A possible reason for using constant‐current stimulation is that the DBS electrode impedance changes over time,^44^ especially in the first 2 to 3 months after surgery.^46^ Once a stimulation protocol has been established and enough time has passed, the preferred technique might be constant‐voltage stimulation, because it lengthens battery life.

### Energy Harvesting

The development of new DBS devices aimed to prolong life and reduce patient discomfort must face two important constraints, size and power consumption. Power consumption is now becoming a less important problem given modern battery technologies and the advent of rechargeable batteries. Also, batteries will in the future most probably give way to energy‐harvesting methods, making implanted devices smaller and avoiding surgery for their replacement. Though energy sources, coming from outside (solar energy, wireless power transfer, infrared radiation) and inside the body (kinetic, thermal) could provide power in the near future, nowadays the best solution for harvesting energy is a wireless transfer through an inductive link.^47^ The need for power delivery to implantable devices and for high‐rate bidirectional communication in brain–computer interfaces is pushing the search for technical solutions into the field of wireless operations.^25^ Future developments in microelectronics will therefore lead to new, smaller DBS devices that consume less power.

## Technology Around the Patient

Technological advances in the DBS field generated around the patient mainly concern the supporting systems designed to improve the quality of care for DBS patients and their caregivers. All of these novel tools are in the mainstream of telemedicine and consumer health informatics and have already proved effective in managing major burden diseases such as cardiovascular diseases^48^ and diabetes.^49^ Consumer health informatics is regarded as the possible bridge between the higher needs for homecare and the expanded responsibility for self‐care and self‐management that patients and families have to bear.^50^

Long‐term DBS outcomes depend on proper monitoring and on recognizing unresponsive PD symptoms or side effects early so that they can be promptly treated.^51^ At home, patients nevertheless rely on a family caregiver, who is usually not trained to deal with PD progression and DBS. The next technological step should be to develop holistic information and communication technology–based systems for DBS home monitoring that would help not only to assess PD progression, but also to make patients and their family central actors in the health care process. Such systems should rely on a strong support for communication and connectivity between patients, families, and reference clinicians, and on neurophysiological biomarkers that could be used to provide alarms for continuous home monitoring and remote patient control from the hospital.^9^ These systems will be implemented on new “apps” running on the patient's commercial smartphone and connected to the clinician's information systems. This integrated scenario is still in its early development stage, insofar as standards and communication architectures, even though envisaged, are still unavailable. We have now proposed and developed a potentially useful tool for this purpose: WebBioBank is a web‐based system for collecting clinical and neurophysiological data, specifically created for DBS management,^52^ that also can be connected to the patient's personal mobile apps and can safely share data.^53^

## Concluding Remarks

A brief look has shown how DBS has been technologically exploited over the past 5 to 10 y. The clinical effects of DBS are now well known, and the numbers of patients treated and the volume of business have progressively increased. Despite these advances, like all commercially appealing technologies, DBS systems have to be constantly modernized. The limitations of conventional DBS have focused clinicians' attention on nonresponders or on difficult patients. One therefore wonders how many patients are non‐responders to conventional DBS. Although the question is difficult to answer and the answer varies among the different indications, for PD approximately one third to one fourth of implanted patients have a suboptimal response to DBS.^4^ Considering the rather large number of patients undergoing DBS, a novel technology could help in numerous cases. Novel technologies also could provide help for possible new indications such as Alzheimer's disease^54^ or depression.^55^ Technology around the patient could really change how we care for patients in all medical fields in the next few years and will probably also change the way we use DBS. With relatively low investments, these cutting‐edge technologies could help large numbers of patients, thus reducing the burden on the national health systems. Although progress in this field is still at the beginning, it is a fascinating new research direction that could bring about a major change in neurological practice.

A potential foreseeable danger is the progressive increase in the cost of DBS devices linked to technological progress. Increasing future costs could limit DBS even more to developed countries, hence the need to reduce costs. Patients and national health systems risk coming to grief because of increasing costs, and health professionals risk being unable to follow patients closely as new‐generation devices become increasingly automated. As clinicians, we should therefore proceed carefully, step‐by‐step, while we ask ourselves whether the novel technological solution really brings health care benefits and carefully assess their cost–benefit ratio, leaving aside the myth about costless progress and mistrusting the idea that new is invariably better than old. These caveats notwithstanding, technological advances should allow clinicians to save the time needed for lengthy and time‐consuming stimulation device setting, thus reducing national health system costs for the procedure. Whether technological progress in DBS will really translate into better patient care awaits confirmation from further research. At the moment, we believe that as more “optionals” became available, postoperative programming might be increasingly complex. Already, most programmers continue using constant‐voltage instead of constant‐current, and few use interleaving or steering, rarely going beyond the accepted average values, stimulation parameters, and electrode configurations. Simulation software and image reconstruction may help, but whether this approach is effective or feasible remains to be demonstrated.

In conclusion, although the galloping technological progress in the DBS field will be important to improve our patients' care, without being left behind in a fast‐moving world, we have to keep abreast of technological DBS developments, avoiding the temptation to waste energy on clinically useless innovations, and always remembering to take a duly cautious, critical, and scholarly approach before accepting their use in our day‐to‐day practice. Advanced technology is of scarce help if it produces little or no clear additional benefit, is expensive, and is too difficult to use.
